# Physiological discrimination and correlation between olfactory and gustatory dysfunction in long‐term COVID‐19

**DOI:** 10.14814/phy2.15486

**Published:** 2022-11-22

**Authors:** Andrea Mazzatenta

**Affiliations:** ^1^ Neuroscience, Imaging and Clinical Sciences Department ‘G. d'Annunzio’ Chieti‐Pescara University Chieti Italy

**Keywords:** ageusia, anosmia, COVID‐19, e‐nose, long‐term COVID‐19, rapid test, smell, taste, taste, VOCs, Volabolome

## Abstract

The spread of the SARS‐CoV‐2 virus produces a new disease termed COVID‐19, the underlying physiological mechanisms of which are still being understood. Characteristic of the infection is the compromising of taste and smell. There is a persistent need to discriminate the dysfunctions and correlation between taste and smell, which are probably epiphenomena of other concealed conditions. Anosmic and ageusic long‐term COVID‐19 patients were re‐evaluated after 1 year using a Volabolomic approach with an e‐nose recording system coupled with olfactometric and gustometric tests. Here a range of sensory arrangements was found, from normal taste and smell to complete losses. The following patterns of olfactory threshold (OT)‐taste threshold‐olfactory uni‐ and cross‐modal perception were found anosmia‐severe hypogeusia‐anosmia; hyposmia‐hypogeusia‐severe hyposmia; normosmia‐ageusia‐hyposmia; severe hyposmia ‐normogeusia‐normosmia. There is a strong correlation between OT and olfactory uni‐ and cross‐modal perception, a moderate correlation between olfactory and taste threshold and no correlation between OT and taste threshold. In conclusion, this study provides evidence for the feasibility of testing the chemical senses to directly objectify function in order to discriminate taste from olfactory impairment. Furthermore, it allows to hypothesize a long‐term effect of the virus due to neuroinvasion through, probably, the olfactory system with injury in the related multisensory areas of taste and smell.

## Graphical abstract

Anosmic and ageusic long‐term COVID‐19 patients were re‐evaluated after 1 year. The following patterns of long‐term COVID‐19 were found: anosmia‐anosmia‐severe hypogeusia; hyposmia‐severe hyposmia‐hypogeusia; normosmia‐hyposmia‐ageusia; severe hyposmia‐normosmia‐normogeusia. A long‐term effect of the virus due to neuroinvasion through, probably, the olfactory system with lesions in the related multisensory areas of taste and smell is therefore conceivable.

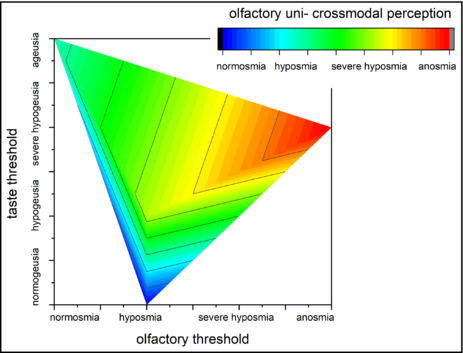

## 1 INTRODUCTION

The current pandemia due to the spread of SARS‐CoV‐2 and its variants produces COVID‐19, which has clinical manifestations ranging from asymptomatic to respiratory failure requiring intensive care unit.

Above all, the characteristic signature of the infection, regardless of variants, is the targeting of chemosensory systems with consequent impairment of the perception of smell and taste (Lechien, Chiesa‐Estomba, De Siati, et al., 2020; Lechien, Chiesa‐Estomba, Hans, et al., 2020; Mazzatenta et al., 2020; Vaira, Salzano, Deiana, et al., 2020). The viral targets Angiotensin‐Converting Enzyme 2 (ACE2) and Transmembrane Serine Protease 2 (TMPRSS2) show extremely high expression in cells characteristic of nasal epithelium (Sungnak et al., 2020), which is the putative starting point of viral neurotropism (for review see Cheng et al., 2020) and cause damage to the olfactory pathway (Mazzatenta et al., 2021).

In addition, ACE2 receptors are widely expressed on the mucosa of the oral cavity, particularly on the tongue (Xu et al., 2020). Application of ACE2 inhibitors and Angiotensin II blockers demonstrate some interaction with taste receptors (Suliburska et al., 2012; Tsuruoka et al., 2005; Zhou et al., 2020). Furthermore, SARS‐CoV‐2 binds the sialic acid receptors, such as the coronavirus Middle East Respiratory Syndrome(Milanetti et al., 2021; Park et al., 2019). Sialic acid, a component of salivary mucin, protects the glycoproteins transporting taste molecules within the taste pores from premature enzymatic degradation (Witt & Miller, 1992). Consequently, a virus‐induced decrease in sialic acid in saliva accelerates the degradation of taste particles and causes an increase in the taste threshold (Pushpass et al., 2019).

Furthermore, cross‐modal flavor perception in these patients is adversely affected by the concomitant presence of olfactory impairments, due to the intimate functional correlation between these chemosensory systems (Capparuccini et al., 2010; Small & Prescott, 2005).

Initially, taste disorders, often based on patients' self‐reported ‘perceptions’, were stated to be more frequent than olfactory impairments (Abalo‐Lojo et al., 2020; Giacomelli et al., 2020; Lechien, Chiesa‐Estomba, De Siati, et al., 2020). Conversely, experimental olfactometric measurements reverse this assumption (Mazzatenta et al., 2020).

Nevertheless, there is a persistent need to discriminate the correlation between the smell and taste dysfunctions and there are probably other factors behind the gustatory disorders in COVID‐19 patients (Capparuccini et al., 2010; Vaira, Salzano, Fois, et al., 2020).

Consequently, we investigated the possibility to evaluate an objective measurement of taste (the ‘Gustometry’) and the correlation with smell impairments in long‐term COVID‐19 patients.

## 2 CASE

One year ago, at the beginning of the SARS‐CoV‐2 infection, 20 subjects (10 of both sexes, aged between 30 and 50 years) were recruited because reporting both anosmia and ageusia. All of them were evaluated for olfactory threshold (OT), by using OST test (Asteria Healthcare; Mazzatenta et al., 2020), and for taste threshold, with a homemade test using supra‐threshold 0.5 g/ml sucrose and sodium chloride, 0.5% of citric acid and quinine. Ethical clearance protocol number colf01.2020 from the Ethics Committee of the Provinces of Chieti and Pescara was acquired. The volunteers provided written in‐formed consent and the procedure was performed in accordance with the ethical standards of the Declaration of Helsinki.

Among these subjects, four recovered normosmia and normogeusia, 12 got severe COVID‐19 with consequent hospitalization and are currently under therapy for several acquired pathologies, which are exclusion criteria from this project. The remaining four subjects (2 of both sexes, f: 35 and 48 years ago, m: 37 and 45 years ago) were recruited in the present work, namely those who had a healthy medical history without any respiratory or sensorial problem, with mild COVID‐19, without hospitalization, and recovered from the disease in approximately 2 months, as established by two consecutive negative molecular swab tests. These subjects were re‐evaluated for OT, taste threshold, uni‐ and cross‐modal olfactory perception by using Volabolomic approach.

Breath pattern and exhaled breath content of volatile organic compounds (VOCs) were continuously measured in a standard controlled condition, in the morning between 10 and 11 a.m. in a VOCs free room that was monitored, before each subject, by means of a control recording the environmental air. In addition, other physical parameters (*T*, brightness) that could affect the VOCs recording were controlled. The recording system used in this experiments was an e‐nose iAQ‐2000 (Applied Sensor) equipped with a metal oxide semiconductor (MOS) having a sensing range of 450–2000 ppm CO_2_ equivalents, which is able to detect a broad range of volatile compounds (both organic and inorganic, e.g., alcohols, aldehydes, aliphatic hydrocarbons, amines, aromatic hydrocarbons, ketones, organic acids, and CO), while correlating directly with the CO_2_ levels (Mazzatenta, Pokorski, & Di Giulio, 2015; Mazzatenta, Pokorski, Montinaro, et al., 2015; Mazzatenta, Pokorski, Sartucci, et al., 2015; Mazzatenta et al., 2016, 2021). The MOS sensor is based on a chemical reaction which occurs between the surface of the sensor and the volatile compounds. This reaction causes the closure of an electrical circuit, which sends a raw electrical signal, in Ohm, to the acquisition system. The signal is recorded, saved, and elaborated by the system algorithm, which expresses the VOCs concentration. The e‐nose was attached to a tight‐sealed mask that completely covered the nose and the exhaled VOCs were measured in real time ad in continuous during normal breath inspiration.

The OT test, based on the Connecticut Chemosensory Clinical Research Center test (Cain et al., 1988), consists of a logarithmic scale, from OT1 to OT9, of decreasing dilution of n‐butanol up to a maximum of 4% and was coupled to e‐nose. The threshold is assigned to the first statistically significant consecutive step. Furthermore, whereas the traditional test requires to interrupt the administration to avoid physiological adaptation when reaching the threshold, because the test is subjective and must be repeated three times, instead, here the use of the e‐nose allowed the subjects to be exposed to the entire set of stimuli from OT1 to 9 in order to compare the responses, only once. Statistical analysis was performed using one‐way ANOVA *p* < 0.05. This test is the most useful for coupling with e‐nose for real‐time measurements. The OT test measures hyperosmia (OT1–2), normosmia (OT3–4), hyposmia (OT5–6), severe hyposmia (OT7–8) and anosmia (≥OT9).

The OT test coupled with the VOC‐recording e‐nose provides, in real time, the objective reproducible, non‐invasive and low‐cost assessment of the olfactory threshold.

The unimodal olfactory stimulus (olf/olf) was measured with e‐nose by using the E‐3,7‐dimetil‐2,6‐ottadienale (IUPAC name, normally called geranial, smelling like orange; CAS number 5392‐40‐5); whereas, cross‐modal paired olfactory stimuli—trigeminal (olf/trig), —gustatory (olf/gus) were recorded by using (R)‐5‐Isopropenil‐2‐metil‐2‐cicloesenone (IUPAC name, normally called R‐(–)‐carvone, smelling like mint; CAS number 99‐49‐0) and ott‐1‐en‐3‐ol (IUPAC name, normally called mushroom alcohol, smelling like mushrooms; CAS number 3391‐86‐4), respectively. Anosmia is represented by no statistical difference with the unstimulated base‐line, normosmia by a significant positive increase over baseline, while hyposmia by a significant negative decrease.

The taste threshold test consists of real‐time e‐nose recording of the response to a 50 μl drop of supra‐threshold stimuli consisting of 0.5 g/ml sucrose or sodium chloride, 0.5% citric acid or quinine, passively released on the middle of the tongue (Mazzatenta et al., 2020). Normogeusia is for significant positive records compared to baseline, ageusia is for no significant difference from baseline, hyposmia and severe hyposmia are related to the degree of impairment (Mazzatenta et al., 2021). Data normalization was made by Log10, treatment and statistical analysis was done by Excel, Origin and Jamovi software (Version 1.6 retrieved from www.jamovi.org) and *α* is set at 0.05.

### 2.1 Olfactory threshold

**FIGURE 1:**
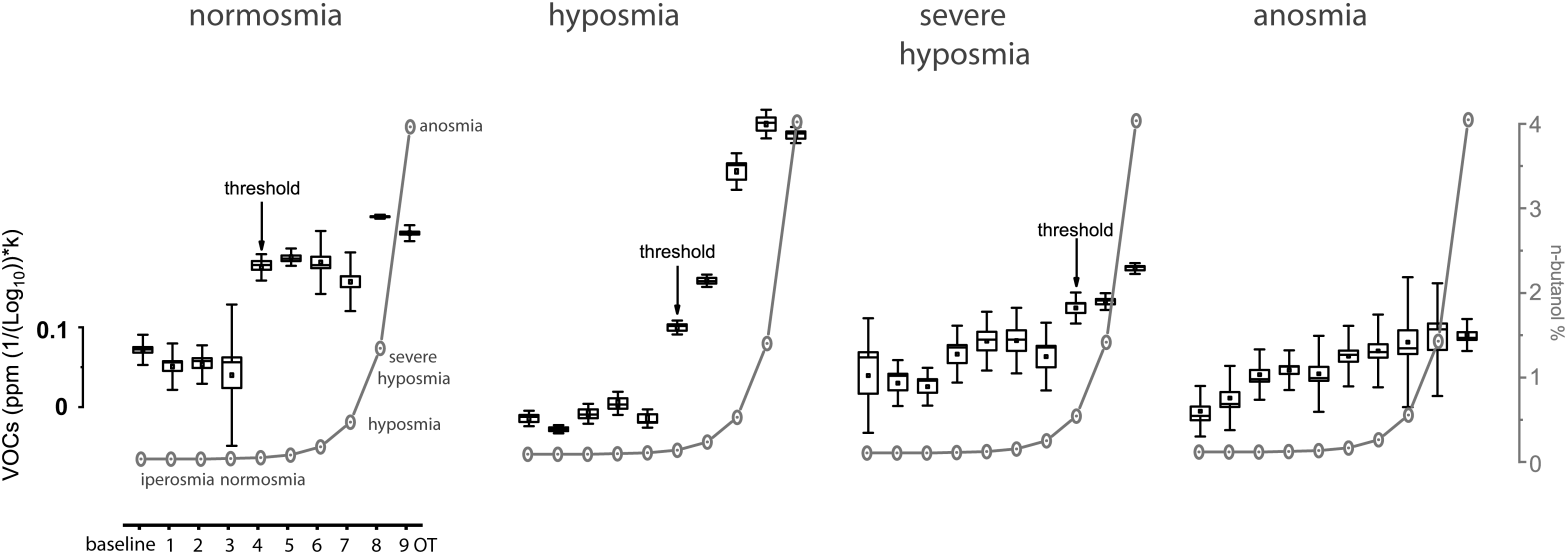
Olfactory threshold recorded by e‐nose in long‐term COVID‐19 subjects. The stimulating steps from OT1 to OT9 are decreasing dilution of n‐butanol to max 4% based on Connecticut Chemosensory Clinical Research Center threshold test (Cain et al., 1988). Threshold is the first statistical significant consecutive step; statistical analysis is carried out by using one‐way ANOVA *p* < 0.05. VOC, volatile organic compound.

OT was measured in subjects who had long‐term COVID‐19 (Figure 1). Statistically, they show an OT ranging from normosmia to anosmia. It should be noted that, unlike the traditional threshold test, here we expose subjects to the entire set (OT1‐9) of n‐butanol dilutions in order to compare responses. In normosmia, a substantial homogeneity of responses was observed up to the OT4 stimulus, which is considered the threshold because it is the first statistically significant consecutive step *p* < 0.05 (*F*_(1,59)_ = 4.8). In hyposmia, the threshold is measured at OT5 *p* < 0.05 (*F*_(1,59)_ = 17), while in severe hyposmia at OT7 *p* < 0.05 (*F*_(1,59)_ = 4.5). In anosmia no statistically significant response was recorded (*p* = 0.66, *F*_(1,59)_ = 0.19), so the threshold is greater than 4% n‐butanol.

### 2.2 Uni‐ and cross‐modal olfactory perception

**FIGURE 2:**
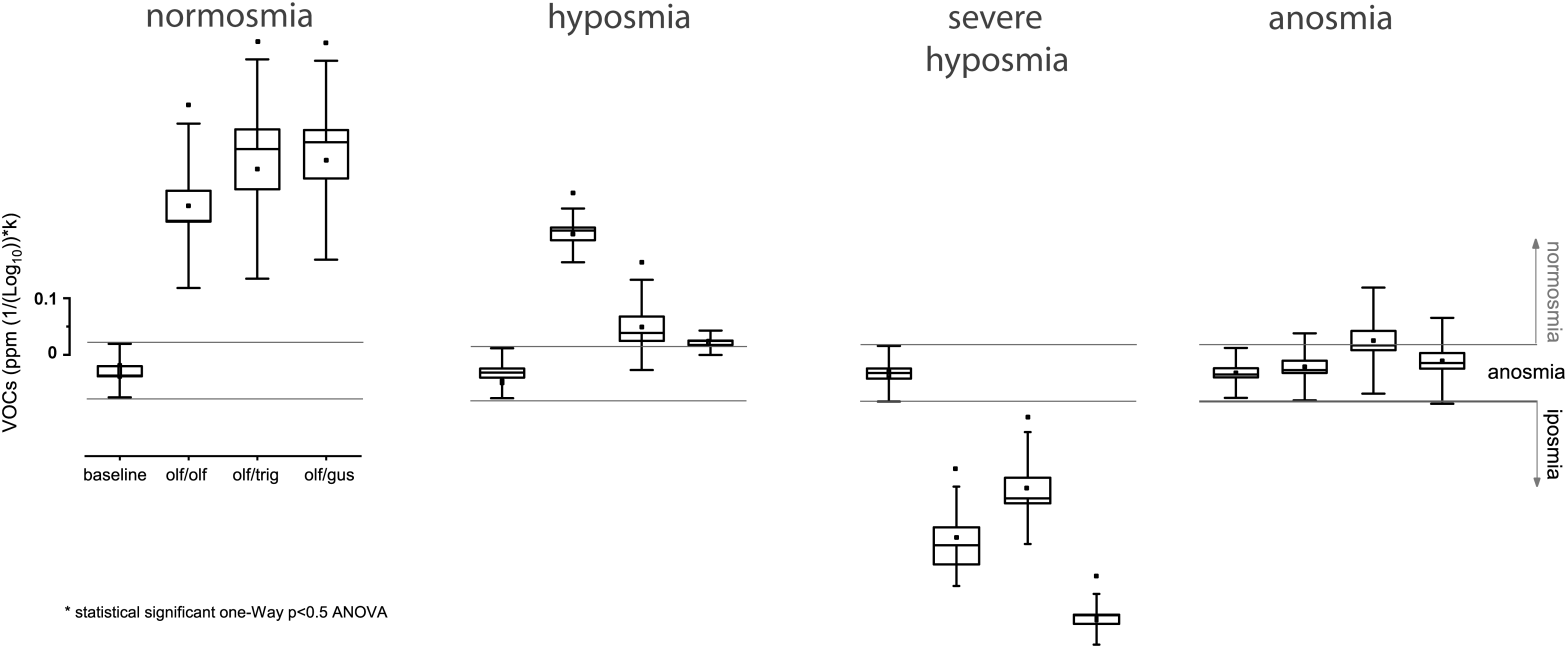
e‐nose uni‐ and cross‐modal olfactory testing. In long‐term COVID‐19 the response to the unimodal olfactory stimulus (olf/olf), cross‐modal paired olfactory stimuli—trigeminal (olf/trig), —gustatory (olf/gus) was recorded. Anosmia is represented by no statistical difference with the unstimulated baseline, normosmia by a significant positive increase over baseline, while hyposmia by a significant negative decrease. Statistical analysis is carried out by using one‐way ANOVA *p* < 0.05. VOC, volatile organic compound.

Responses to uni‐ and cross‐modal olfactory stimulation were investigated in long‐term COVID‐19 subjects. These varied from normosmia to anosmia. In normosmia, all three stimuli returned a statistically significant ‘positive’ response compared to baseline (baseline vs. olf/olf *p* < 0.05, *F*_(1,59)_ = 55.3; baseline vs. olf/trig *p* < 0.05, *F*_(1,59)_ = 1283; baseline vs. of/gus *p* < 0.05, *F*_(1,59)_ = 583.4). Whereas in hyposmia uni‐modal olfactory and cross‐modal trigeminal perception were significant (baseline vs. olf/olf *p* < 0.05, *F*_(1,59)_ = 256.7; baseline vs. olf/trig *p* < 0.05, *F*_(1,59)_ = 97.4) while the cross‐modal olf/gus stimulus was not statistically significant compared to baseline. In severe hyposmia the responses are ‘negative’ and significant compared to baseline (baseline vs. olf/olf *p* < 0.05, *F*_(1,59)_ = 119; baseline vs. olf/trig *p* < 0.05, *F*_(1,59)_ = 100.8; baseline vs. of/gus *p* < 0.05, *F*_(1,59)_ = 133.1); in anosmia no statistical difference is measured (Figure 2).

### 2.3 Taste threshold

**FIGURE 3:**
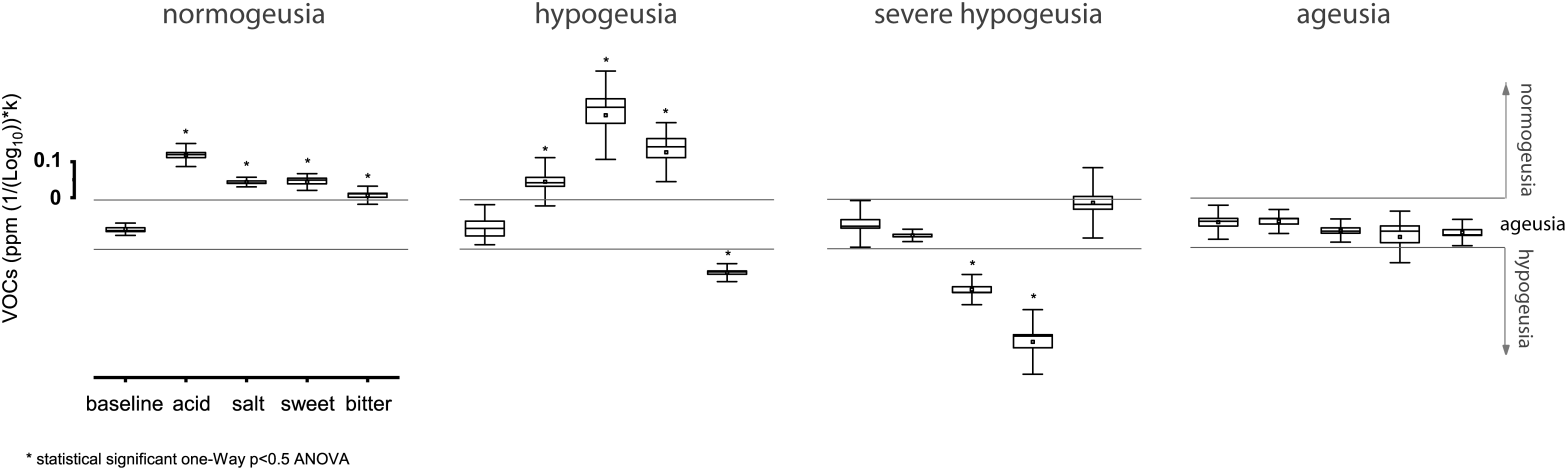
e‐nose taste threshold test in long‐term COVID‐19. Stimuli were supra‐threshold 0.5 g/ml sucrose and sodium chloride, 0.5% citric acid and quinine. Normogeusia is for significant positive records compared to baseline, ageusia is for no significant difference over baseline, hypogeusia and severe hypogeusia are related to the degree of impairment. VOC, volatile organic compound.

Responses to supra‐threshold sucrose 0.5 g/ml or sodium chloride, citric acid 0.5% or quinine stimulation were studied in long‐term COVID‐19 subjects. The e‐nose recorded responses, ranging from normogeusia, which is for significant positive recordings compared to baseline (baseline vs. acid *p* < 0.05, *F*_(1,59)_ = 181.2; baseline vs. salt *p* < 0.05, *F*_(1,59)_ = 159.9; baseline vs. sweet *p* < 0.05, *F*_(1,59)_ = 87.6; baseline vs. bitter *p* < 0.05, *F*_(1,59)_ = 16.1), to ageusia, which consists of no significant difference from baseline (*p* ≥ 0.05). Hypogeusia (baseline vs. acid *p* < 0.05, *F*_(1,59)_ = 6.9; baseline vs. salt *p* < 0.05, *F*_(1,59)_ = 565.8; baseline vs. sweet *p* < 0.05, *F*_(1,59)_ = 87.6; baseline vs. bitter *p* < 0.05, *F*_(1,59)_ = 35.3) and severe hypogeusia (baseline vs. acid *p* = 0.92, *F*_(1,59)_ = 0.011; baseline vs. salt *p* < 0.05, *F*_(1,59)_ = 195.8; baseline vs. sweet *p* < 0.05, *F*_(1,59)_ = 52.1; baseline vs. bitter *p* < 0.05, *F*_(1,59)_ = 31.9) are negatively correlated with baseline and degree of impairment (Figure 3).

### 2.4 Discrimination and correlation analysis in long‐term COVID‐19

**FIGURE 4:**
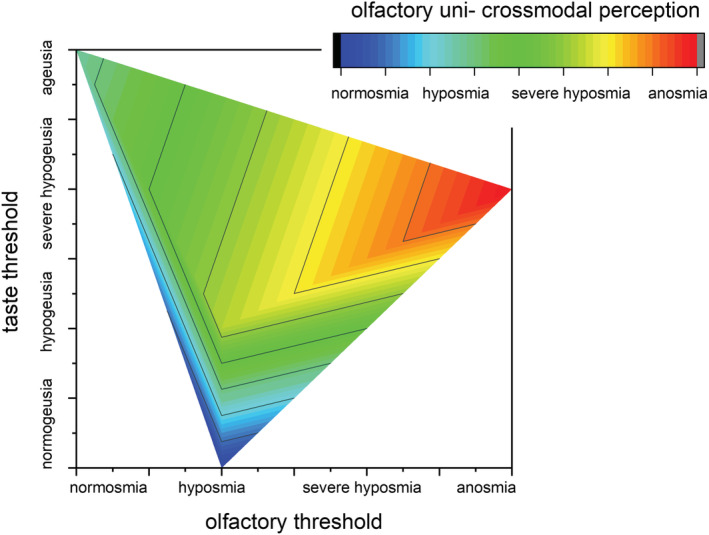
Correlation between Olfactometry and Gustometry in long‐term COVID‐19. The emerging correspondences are: Anosmia‐severe hypogeusia‐anosmia; hyposmia‐hypogeusia‐severe hyposmia; normosmia ‐ageusia‐hyposmia; severe hyposmia‐normogeusia‐normosmia.

To better characterize the long‐term effects of SARS‐CoV‐2 on chemical senses we correlate the Olfactometry to Gustometry (Figure 4). The resulting picture refers to: (i) a complete olfactory anosmia coupled to severe hypogeusia; (ii) OT hyposmia and taste hypogeusia paired to cross‐modal severe hyposmia; (iii) OT normosmia and taste ageusia combined to cross‐modal hyposmia; (iv) OT severe hyposmia and taste normogeusia joined to cross‐modal normosmia. Pearson's *r* coefficient = 0.718 shows a highly positive correlation between OT and olfactory uni‐ and cross‐modal perception; while scarce correlations (*r* = 0.4) is observed between olfactory perception and taste and no correlation (*r* = −0.1) is observed between OT and taste.

## 3 DISCUSSION

This study investigated the OT, taste threshold, uni‐modal and cross‐modal olfactory perceptions recognized as hallmark symptomatology in the infection (Spinato et al., 2020).

The results achieved in this study point to (i) olfactory and taste dysfunction characterizes COVID‐19; (ii) characteristic of long‐term forms; (iii) Strengthens the SARS‐CoV‐2 neuroinvasion hypothesis.

In this study, long‐term COVID‐19 patients, the 20% of the original COVID‐19 group, sampled with complete anosmia and ageusia, was re‐tested 1 year later because the subjects still had dysfunction in smell or taste or both, putatively ascribable to long‐term COVID‐19 (Wang et al., 2020; Yelin et al., 2020). In 20% of them, a complete recovery of normosmia and normogeusia was achieved, which we can define as post‐COVID‐19, the remaining subgroup had severe disease and still had a number of related disorders according to previous literature (Carfì et al., 2020; Gorna et al., 2021) and was therefore excluded and defined as Post‐Hospitalization COVID‐19 (PHosp‐COVID‐19).

Among the long‐term COVID‐19 patients we studied OT, taste threshold, uni‐ and cross‐modal perception, their discrimination and correlation. Because taste and smell are closely related, it is believed that most cases in which taste is lost are due to damage in the olfactory system (Soter et al., 2008; Stinton et al., 2010). The gustatory system is not usually selectively affected by viral upper respiratory tract infections (Bromley, 2000; Rolls, 2019; Small et al., 1892–1903). Except for one observational study (Tarifi et al., 2021), to the best of our knowledge, no olfactometric and gustometric studies have been carried out on long‐term COVID‐19 patients, neither correlation nor discrimination. Consequently, this study aimed to measure the variability, discrimination, and correlation between olfactory and taste among long‐term patients with persistent olfactory and gustatory dysfunctions.

Regarding variability, a wide range of responses from normosmia to anosmia and from normogeusia to ageusia was measured. Taken on their own these results are not informative at all and they seem quite confusing. In fact, in clinical practice, a single patient showing, for example, normosmia‐hyposmia‐ageusia for the olfactory, perception, and taste threshold, respectively, is puzzling.

However, looking at these results from a physiological perspective and correlating their significance, the revealing picture is completely different. Indeed, a strong correlation was found between OT and perception, which is due to the physiological significance of these two parameters, according to Haddad, Khan, et al. (2008) and Haddad, Lapid, et al. (2008) As the sense of smell is represented by a multidimensional space, threshold, uni‐ and cross‐modal perception are the most important parameters that enable discrimination and identification and influence memory, both short‐ and long‐term, along with Hedner et al. (2010) and Kermen et al. (2011) Consequently, the threshold ‘serves’ for perception since Fechner's (1966) definition and its subsequent revisions (Rouder & Morey, 2009).

In this respect, the lack of correlation between OT and taste is in line with the literature outcome (Doty & Crastnopol, 2010; Thumfart et al., 1980). Functionally they appear as unrelated parameters.

However, it is clear that information about the surrounding milieu is typically obtained from more than one sensory system, requiring our brain to integrate multi‐modal stimuli into a coherent sensorial experience. This is particularly important in the human sense of smell, a system that is highly dependent on the support of multi‐sensory inputs, especially taste (Zhou et al., 2019). For example, in rodents, gustatory stimuli have been shown to modulate the activity of individual units in primary olfactory areas (Rolls et al., 1989); and tastants applied to the tongue induce responses in prefrontal cortex (Maier et al., 2012). In humans, functional magnetic resonance imaging studies have shown that visual and auditory stimuli, that are related to odors, can activate prefrontal cortex (Ripp et al., 2018; Zhou et al., 2019).

According to the above studies, we can observe the partial correlation between the perceptions of smell and taste, which are correlated information. In humans, multi‐sensory convergence and integration is based not only on the classical hierarchical multisensory areas, but also on multisensory integration involving a distributed network with multiple redundant pathways (Ghazanfar & Schroeder, 2006; Maier et al., 2015).

For example, the olfactory bulb, rectus gyrus, posteromedial portion of the frontal lobe with its medial olfactory areas, amygdaloid nucleus, hippocampal gyrus is among the structures involved in many forms of olfactory pathology (Schellinger et al., 1983). Similarly, the posterior thalamus acts as the relay station for gustatory stimuli and is involved in taste dysfunction (Schellinger et al., 1983). However, combined taste and smell loss involves several brain areas, such as the orbitofrontal cortex, frontal and/or anterior temporal lobe and suggests a diffuse taste and smell pathway in the brain (Iannilli & Gudziol, 2019; Rolls, 2004). This result strengthens the hypothesis of SARS‐CoV‐2 neuroinvasion (Pezzini & Padovani, 2020).

In conclusion, objective evaluation of smell and taste are relevant to detect long‐term impairment of chemical perceptions and possible involvement of central areas.

## AUTHOR CONTRIBUTIONS

A.M. curate all aspect of the paper.

## Funding information

No funding information provided.

## CONFLICT OF INTEREST

The author declares no conflict of interest.

## ETHICS STATEMENT

Ethical clearance protocol number colf01.2020 from ‘Comitato Etico delle Province di Chieti e di Pescara’ has been obtained.
